# Anatomy Bill. Mr. Guthrie's Lectures

**Published:** 1830-04-01

**Authors:** 


### ANATOMY BILL. MR. GUTHRIE'S LECTURES.

In a late lecture at the College of Surgeons, Mr. Guthrie took occasion to dwell at considerable length on the subject of dissection, and the difficulties thereon attendant. We find that Mr. G. is of the same opinion with ourselves, as to the supposed stigma of murderers being given up for dissection. We verily believe that this stigma has not the slightest operation in generating a horror of anatomy in this or in any country. The following is unanswerable.

"In Ireland, I believe, those invested with judicial functions well know, that when a man is ordered to be executed, his nearest relations entreat those functionaries, in the most earnest manner, in order to prevent the body from being given to dissection. In no country in the world is dissection more freely and more fairly carried on than it is at this moment in the city of Dublin. Whilst it is endeavoured to be instilled into the minds of the profession here, that it is because murderers are dissected that subjects are not to be procured, we find that the profession in Dublin take an opposite course. We find that they are fully convinced and satisfied that it has no influence upon the public mind. We find that there is no place where bodies are obtained so readily (they are obtained almost for nothing); —there is no fear of dissection; they are capable of doing as they please;— yet we find them actually selecting the bodies of murderers—not only seeking them for dissection, but publishing, in the face of the whole country, the experiments performed upon them. But if any ground of suspicion that the prejudice depended on the anatomizing of murderers were entertained, this would be most indiscreet, and might put a stop to the freedom of dissection which at the present moment prevails in Dublin. We find, on the contrary, that they not only open these subjects, but perform experiments upon them, and publish the result in the newspapers. We see that they are not afraid of these descriptions going forth, and we may therefore reasonably and fairly suppose that there is an error upon this subject."

In a celebrated article in the Quarterly Review by the late Dr. Gooch, it is said—"let the profession refuse to dissect the bodies of murderers, and throw that obstacle in the way of the laws:—let them refuse to do what they are called upon to do, and it must be abandoned." But it is to be remembered that the charter of the College of Surgeons is held by the tenure that they shall find a place for the dissection of murderers. We believe that the abolition of the said law would do nothing for the removal of the difficulties under which practical anatomy now labours—nor would its abolition be attended with any inconvenience.

Mr. Guthrie has passed a sharp criticism on the defective points in the late bill, and has shewn, in a very clear manner, the grounds on which the College of Surgeons raised their objections to its enactment as a law. As we know, from tolerably good authority, that either the bill of Mr. Warburton will not be presented at all, or, if presented, that it will be divested of its objectionable clauses, we shall not enlarge on the defects, which Mr. Guthrie has so forcibly criticised. The principal grounds of the College opposition to the bill were—the majority of non-professional commissioners—(an enactment that indicated a distrust of the honorable conduct of the medical commissioners)—the imposition of a tax of 5 guineas upon every person who taught publickly the art and science of anatomy —(a tax that subjected a private surgeon to a penalty for improving himself in anatomy by private dissection, unless he took out a license for such dissection)—and, lastly, the clause requiring interment of the body dissected—an impossibility under ordinary circumstances. Mr. Guthrie taxed the late bill, in pretty strong terms, as being liable to the imputation of saying things which it did not mean, and meaning things which it did not say. We believe Lord Althorpe means to bring in a bill simply to legalize the sale of dead bodies, unclogged with restrictions and technicalities.

The remaining lectures on anatomy and physiology were, lecture 2, on the anatomy of the bones of the pelvis, not only with relation to the differences between the sexes, but on the peculiarities and capacities of the female pelvis, and on the different motions of the head of the fœtus during the parturition. The views of Cowper and others, in relation to the gradation of the form of the head from man down to the monkey were noticed, and the principle applied to the pelvis; the differences of form, shape, and structure between the male and female of the European, as compared with the African, the Hottentot, the East Indian, and down to the Ourang Outang, were demonstrated, and the gradation was then carried through several races of animals, from the common climbing bear to the polar bear, and down to the marsapial animals, such as the kangaroo. The peculiarities attendant on the use of their bones were noticed, and the whole illustrated by numerous diagrams and drawings.

The third, fourth, fifth, and sixth lectures were on the anatomy of the kidneys, ureters, and bladder in man and in animals; the differences in situation between the soft parts in the male and female pelvis:—On the anatomy of the urethra and external parts, and on the introduction of instruments into the bladder.

